# Enhancing credit scoring accuracy with a comprehensive evaluation of alternative data

**DOI:** 10.1371/journal.pone.0303566

**Published:** 2024-05-21

**Authors:** Rivalani Hlongwane, Kutlwano K. K. M. Ramaboa, Wilson Mongwe

**Affiliations:** 1 Graduate School of Business, University of Cape, Cape Town, South Africa; 2 Electrical and Electronic Engineering, University of Johannesburg, Johannesburg, South Africa; JNKVV: Jawaharlal Nehru Krishi Vishwa Vidyalaya, INDIA

## Abstract

This study explores the potential of utilizing alternative data sources to enhance the accuracy of credit scoring models, compared to relying solely on traditional data sources, such as credit bureau data. A comprehensive dataset from the Home Credit Group’s home loan portfolio is analysed. The research examines the impact of incorporating alternative predictors that are typically overlooked, such as an applicant’s social network default status, regional economic ratings, and local population characteristics. The modelling approach applies the model-X knockoffs framework for systematic variable selection. By including these alternative data sources, the credit scoring models demonstrate improved predictive performance, achieving an area under the curve metric of 0.79360 on the Kaggle Home Credit default risk competition dataset, outperforming models that relied solely on traditional data sources, such as credit bureau data. The findings highlight the significance of leveraging diverse, non-traditional data sources to augment credit risk assessment capabilities and overall model accuracy.

## Introduction

The rise of big data has fuelled the use of machine learning techniques in credit scoring [1]. However, big data often exhibits complex nonlinear relationships and high dimensionality (for example, numerous predictor variables [2]). These characteristics pose challenges for traditional modelling methods like logistic regression and linear discriminant analysis, which may struggle to yield accurate predictions in this context [2–5].

Beyond its inherent complexity, big data offers advantages in credit risk scoring through the incorporation of alternative data sources [6]. Research is increasingly exploring the potential of alternative data to improve credit risk prediction accuracy, particularly for individuals lacking traditional credit histories [7,8]. Studies suggest that including alternative data could expand credit access to millions in the United States alone [7]. This number is likely even higher in developing countries. By leveraging alternative data, banks can broaden their customer base to include those with limited or no conventional credit history [7].

A critical research gap exists in systematically analysing the impact of excluding alternative data from credit scoring models. This omission limits our understanding of the consequences for individuals lacking comprehensive credit bureau data, a challenge further amplified by privacy concerns surrounding such data [7,9]. To address this gap, this study investigates the effects of removing alternative data attributes, such as social context and regional evaluations, from predictive models. By assessing the significance of these alternative data sources, our research aims to inform credit assessment mechanisms that can integrate individuals with limited or no traditional credit history.

This study addresses a gap in the literature by employing the model-X knockoffs variable selection framework within credit scoring. This framework’s suitability for high-dimensional datasets makes it particularly valuable when examining the Kaggle Home Credit data [10]. By utilizing the model-X knockoffs approach, our study advances upon prior research that did not leverage this method for predictor variable selection.

This study leverages the Kaggle Home Credit dataset, a comprehensive resource for credit risk assessment containing information on 356,255 individuals. The dataset encompasses diverse data types relevant to credit scoring, including application details (age, income, city rating, residency, employment, dependents, social network credit defaults), credit bureau data (loan delinquency, credit types held), and loan details (credit limits, payment history, outstanding balances) [11]. This multifaceted dataset captures various dimensions of borrowers’ financial profiles, making it valuable for analysing the impact of alternative data on creditworthiness assessment.

Prior research on the Kaggle Home Credit dataset has demonstrated the effectiveness of tree-based algorithms for credit risk modelling [11–16]. Notably, [11] achieved a strong benchmark with their LightGBM model. This study builds upon this work, introducing a novel predictor variable selection approach based on the model-X knockoffs framework. This approach achieves a higher AUC than prior methods, including the feature removal approach utilized in [11].

This paper is structured as follows: The Literature Review section presents a review encompassing models constructed using the study data and alternative data sources. The Methods section outlines the employed modelling techniques. In the Research Design section, we detail the research design of the study. The Results and Discussion section covers the results and discussion. Finally, the Conclusion section concludes with a summary of findings and outlines avenues for future research.

## Literature review

This section explores themes related to the use of alternative data in credit scoring. It examines various alternative data such as psychometric, social networking and telecommunication. Furthermore, a comprehensive review of previous research specifically examining studies conducted by other researchers who used the same dataset employed in our research.

### Alternative data

Credit scoring models are usually built using demographic and predictor variables derived from the historical performance of credit accounts [17]. In the current era of big data characterised by diverse data from sources such as mobile telecommunication companies, Internet of Things (IoT) devices, wearables, social networks, and smartphones [6,18], there is a shift toward leveraging the benefits of big data to incorporate alternative data into credit scoring models [19]. Big data introduces alternative data sources that were previously overlooked by banks in credit scoring. Examples of alternative data include psychometric indicators (e.g., assessing if a credit applicant is a team player), email data (analysing patterns like fractions of emails sent on specific days of the week), social media insights (considering the willingness to share details of a social media account), mobile phone-related details (such as the duration since the phone was changed), and telecommunication data (including call detail records with information on call or SMS origin, destination, duration, date, cost, and mobile carrier) [17,20,21]. This shift toward alternative data signifies a broadening scope for credit scoring models.

In the study by [17], it was shown that augmenting demographic data with alternative data, including psychometric and email predictor variables, led to an enhancement in model performance, measured by the area under the curve (AUC). Moreover, the research carried out by [20] revealed that credit scoring models using call detail records (CDRs) outperformed those relying on credit bureau predictor variables in terms of predictive accuracy, as indicated by the AUC. In [21], the impact of social media predictor variables, such as data on social media presence from a popular platform, borrowers’ voluntary disclosure of their social media accounts, and their activities on social media, was found to be predictive of borrowers’ default probability. The inclusion of social circles-related predictor variables, a less explored area in research, gained significance in a study by [22], emphasizing the importance of customers within a social network.

Researchers such as [6,17], have demonstrated that alternative data such as psychometric, emails, and telecommunications records can be used to complement existing data sources to build more accurate credit scoring models. Furthermore, besides enhancing the performance of credit scoring models, researchers such as [6,20] have suggested that alternative data can independently be employed to construct credit scoring models. This approach, as emphasized by [23], is advantageous as it enables the scoring of individuals without historical performance data from a credit bureau, thereby enabling these customers to access credit.

Even though alternative data has been shown to be predictive in credit scoring, it does have its shortcomings such as not being readily available due to privacy concerns [24], and the data is vulnerable to manipulation and bias due to individuals that associate themselves with a select group of individuals to boost their credit scores [18]. Additionally, acquiring psychometric data poses challenges, as it requires interviews conducted by experienced credit analysts, making it a time-consuming process that is difficult to scale [25].

In the following sections, we explore each type of alternative data that has been previously investigated by other researchers in the context of credit scoring.

### Psychometric and email data

The research by [26] demonstrated that psychometric data can predict loan repayment, with impatience being linked to loan default. Previous studies [25,27] examined the effectiveness of psychometric data in evaluating credit risk for Peruvian entrepreneurs, revealing its utility for individuals lacking credit bureau information. However, the utilization of psychometric data necessitates time-consuming interviews with applicants, posing scalability challenges for large banks.

The study by [17] investigated email predictor variables in credit risk prediction, identifying certain email usage patterns and psychometric predictor variables as effective predictors. Notably, [17] revealed that this dataset exhibits non-linear characteristics, presenting challenges for traditional approaches like logistic regression. In contrast, neural networks demonstrate superior accuracy in capturing the complex, non-linear relationships inherent in the data.

### Social networking data

Researchers have investigated social media data and its potential to expand credit access, particularly for individuals with limited traditional credit history. One notable approach is "social scoring", as proposed by [18], which utilizes social data to evaluate creditworthiness by assessing individuals’ social networks with similar credit scores. In predicting loan default, [21] found significant influence from social media attributes, particularly on Weibo, using logistic regression analysis.

The study conducted by [28] cantered on sentiment analysis of Twitter data for credit ratings but found it to be less predictive than financial ratios. In predicting individual sentiment towards banks using Twitter data, [29] employed a combination of algorithms with financial and credit scores to achieve higher accuracy, although this approach may not be suitable for customers without credit scores.

Furthermore, [22] explored Facebook data obtained from LenddoEFL and identified individuals with similar interests and preferences, referred to as Look-a-likes (LALs) [24]. The study demonstrated that LALs exhibit predictive capabilities for credit risk when utilizing logistic regression and linear support vector machine (SVM) models.

Despite the promise of social media data, limitations include restricted access to social networking sites in certain countries, reluctance of customers to provide data access, and some individuals lacking social media accounts [30].

### Telecommunications data

Early research, such as [20], utilized Call Detail Records (CDRs) to predict default, discovering that advanced machine learning techniques, like Gradient Boosted Trees, outperformed traditional methods such as logistic regression on credit bureau data [20]. Subsequent studies, like [31], further explored the potential of CDR data. While [31] focused on socio-behavioural attributes using XGBoost, [6] employed graph theory to construct social networks and various models. Both studies confirmed the value of CDR data in credit scoring, complementing existing data sources and predicting default [6]. Moreover, [32] examined the impact of telecommunication data with a smaller sample, finding that the random forest model produced the best performance. Despite being a viable data source, models on smaller datasets underperformed [32].

The advantages of telecommunication data lie in its broader accessibility and ease of use compared to other sources. In addition to the research highlighted above on using telecommunication data in credit scoring, studies have demonstrated its predictive power for behaviour and personality traits, aiding targeted marketing [33,34]. However, privacy concerns persist [25,27,30].

In the next section, we review the literature on feature engineering, examining various approaches, including data transformations and aggregations to enhance credit scoring models.

### Feature engineering

The study by [35] describes feature engineering as a process of creating new predictor variables from existing data to provide useful insights. One approach to achieve this is by transforming or aggregating existing predictor variables [36]. In the study by [15], which utilized the Kaggle home credit data, data aggregation techniques (for example, count, sum, average) were employed to enhance the dataset. Specifically, individual records were grouped by client ID, and transactions were summarized, resulting in an increase in the number of predictor variables. However, the study [15] does not indicate the exact number of additional predictor variables created through this process. In contrast, the study by [16] did not employ any feature engineering on the data, and consequently, the models were developed using the original set of predictor variables without any alterations. Notably, the study [15], which performed feature engineering, achieved higher predictive accuracy in credit scoring models compared to [16].

In the study by [37], feature engineering methods were proposed to develop credit card fraud detection techniques. The study utilized aggregations such as count, average, and sum to create predictor variables based on specified elements, such as the aggregation period and behavioural measures. For example, using elements like the last week’s purchase transactions and transaction time intervals, the resulting variable could represent the average time interval between successive purchase transactions within the last week [37]. The study demonstrated that feature engineering is an effective and feasible mechanism for credit fraud detection.

While aggregation is an effective technique for creating additional predictor variables, the challenge lies in determining the appropriate transaction aggregation period [37]. Different time intervals may capture diverse patterns and trends in the data, thereby influencing the effectiveness of the aggregated variables [37].

In the next section, we examine literature on feature selection, crucial in navigating the challenges posed by large volume of data, focusing reducing predictor variables for improved model performance.

### Feature selection

With the increase in the availability of data, a crucial consideration in model development is the reduction of predictor variables [38]. This reduction offers several benefits, including enhanced learning speed during model training, reduced model complexity, and improved generalization capacity and accuracy [38]. Furthermore, it is important to address the impact of high correlation among predictor variables, which can lead to challenges related to multicollinearity, potentially affecting the stability and interpretability of models [39]. However, it is essential to acknowledge that removing predictor variables from the dataset may result in information loss [40].

Feature selection is a common technique used to select predictor variables for modelling purposes without losing significant information [38]. To enhance credit scoring predictions, [41] indicated that feature selection techniques such as information gain, gain ratio, and chi-square have been employed in their research. Similarly, the study by [42] utilized the neighbourhood rough set (NRS) for feature selection, evaluating multiple feature selection algorithms to improve accuracy on two credit datasets. Additionally, in [43], a novel hybrid ensemble credit scoring model that combines five feature selection algorithms is proposed. A recurring pattern in these studies is the presence of a relatively small number of predictor variables in the experimental data, typically fewer than a hundred.

Previous research utilizing the same dataset as our study, such as [11,15], encountered the challenge of managing hundreds of predictor variables. In the study by [11], predictor variable selection involved retaining variables with missing values falling below specified thresholds at various proportions. However, the methodology employed for predictor variable reduction, if any, was not explicitly detailed in the study by [15]. It was through the work of [10] that a novel approach was introduced to address high-dimensional data in credit risk assessment, using the same dataset as [11,15]. This approach involved a high-dimensionality-trait-driven learning paradigm encompassing data categorization, trait-driven feature extraction, and model selection. Experiments conducted on two credit datasets confirmed the effectiveness of this paradigm, providing valuable practical insights for financial institutions.

## Methods

This study focuses on XGBoost, LightGBM, and CatBoost for several reasons. Prior research has extensively demonstrated the effectiveness of tree-based methods in credit scoring, particularly their ability to handle nonlinear relationships and complex interactions typical of credit scoring data [11,44,45]. By focusing on these algorithms, this research aims to investigate the potential of alternative data and the model-X knockoffs framework for variable selection within this established modelling paradigm.

### XGBoost

In an XGBoost model, individual decision trees are sequentially trained. The primary objective of boosting is to improve the performance of previously constructed decision trees [46]. At each iteration, XGBoost places greater emphasis on misclassified data points by assigning higher weights to these samples, guiding the training of subsequent trees. Additionally, XGBoost incorporates regularization techniques to manage model complexity, thereby preventing overfitting and improving generalization to unseen data [46].

In their study, [47] conducted a comprehensive experiment to benchmark various classifiers, including logistic regression, neural networks, support vector machines, random forest, and XGBoost, using credit data. Their analysis revealed that XGBoost achieved the highest accuracy among the examined techniques. Furthermore, to bridge the gap between research and practical implementation, they compared their results to the well-established Fair, Isaac and Company (FICO) credit scores—the industry benchmark for consumer risk assessment in the U.S. Surpassing even the widely accepted FICO scores, XGBoost demonstrated its superiority as a credit scoring model.

### LightGBM

Similar to XGBoost, LightGBM is also a gradient boosting technique [15]. The main distinction between the two lies in their tree growth strategies. LightGBM selects the leaf with the maximum gain during tree growth, leading to a more depth-first approach [15]. In contrast, XGBoost adopts a breadth-first approach to tree growth [15]. Consequently, LightGBM tends to be faster than XGBoost [15].

In prior studies (for example, [11,48]), LightGBM has demonstrated its efficacy as a modeling technique in credit scoring. Notably, in [46], LightGBM outperformed alternative methods, including XGBoost, CatBoost, support vector machines, and logistic regression, in terms of predictive accuracy. Boosting techniques like LightGBM are well-suited for managing high-dimensional data [48]. Adding more trees to the model can enhance predictive accuracy, however it leads to increased model complexity and longer computation times, as highlighted in [48].

### CatBoost

Similar to XGBoost and LightGBM, CatBoost is also a gradient boosting technique [12]. However, CatBoost distinguishes itself from XGBoost and LightGBM by its seamless handling of categorical variables [12].

In prior studies (for example, [12,49]), CatBoost has been shown to outperform other tree-based methods. Specifically, in [49], CatBoost demonstrated superior accuracy compared to techniques such as LightGBM, XGBoost, logistic regression, support vector machines, and random forests across diverse credit datasets. Similar to other tree-based methods like XGBoost and LightGBM, there is a trade-off of increased computational time during training when increasing the number of trees in model fitting.

### Model-X knockoffs

The Model-X knockoffs framework offers a robust approach for variable selection in high-dimensional datasets [50]. It addresses the issue of false discoveries (FDR) by creating "knockoff" variables that mirror the relationship between original features and the target, ensuring the identification of genuinely relevant variables [51]. Unlike earlier knockoff methods limited to linear models [52,53], the framework’s flexibility, particularly the deep knockoffs method, allows it to handle complex, non-linear datasets [54]. This makes it applicable across various fields. While computationally demanding, deep knockoffs are valuable when the number of variables is large [54]. Importantly, controlling FDR is crucial in variable selection, especially in high-dimensional settings [51].

Additionally, while methods like the information gain can highlight important variables, they do not directly control for false discoveries, underscoring the unique value of the Model-X framework’s FDR control.

Research on Model-X knockoffs often focuses on genome-wide association studies [55–57], where its ability to control FDR makes it ideal for identifying genes with a true association to a given trait [55–57]. This framework has been shown to outperform alternative methods in this context.

## Research design

In this section, we cover the approach used for the development and assessment of credit scoring models in this study. This approach covers key phases, including data preprocessing, feature engineering, variable selection using the model-X knockoffs framework, scaling of predictor variables, fine-tuning of hyperparameters, and the assessment of performance metrics for model evaluation.

### Data

This study analyses 356,255 customers who obtained home loans, with the data sourced from [19]. Among them, 24,845 customers are classified as bad due to defaulting on their home loan accounts. The dataset comprises credit bureau, alternative, and demographic data. The primary focus of this study is to evaluate the effect of alternative data (including applicant’s external scores, family, social circles, and geographical variables) on model performance. Additionally, credit bureau and demographic data are examined, as these variables are commonly employed in the development of credit scorecards [19]. Notably, 30% of customers are not in the credit bureau, showing a lower bad rate (6.78%) compared to customers within the bureau (7.05%), resulting in an overall bad rate of 6.97% and a good rate of 93.03%. The target variable indicates whether a customer is in default or not.

Table 1 provides a view of the alternative predictor variables. These predictor variables capture social, geographic, financial, and behavioural information that is distinct from the data directly sourced from the credit bureau’s records.

**Table 1:** Alternative predictor variables.

Predictor variable	Description	p-value
CNT_CHILDREN	Number of children the client has	0.00004
CNT_FAM_MEMBERS	The client’s family size	0.00000
OBS_30_CNT_SOCIAL_CIRCLE	The number of instances in the client’s social surroundings with observed 30 days past due (DPD) default	0.00000
DEF_30_CNT_SOCIAL_CIRCLE	The number of instances in the client’s social surroundings with observed 30 DPD default	0.00000
OBS_60_CNT_SOCIAL_CIRCLE	The number of instances in the of client’s social surroundings with observed 60 DPD default	0.00000
DEF_60_CNT_SOCIAL_CIRCLE	The number of instances in the client’s social surroundings with observed 60 DPD	0.00000
DAYS_LAST_PHONE_CHANGE	The number of days before the application when the client changed their phone.	0.00000
REGION_RATING_CLIENT_W_CITY	Rating of the region where client lives taking city into account (1,2,3)	0.00000
REGION_RATING_CLIENT	Rating of the region where client lives (1,2,3)	0.00000
REGION_POPULATION_RELATIVE	Normalized population of region where client lives (higher number means the client lives in more populated region)	0.00000
REG_CITY_NOT_LIVE_CITY	A flag of whether the client’s permanent matches the contact address (1 = different, 0 = same, at city level)	0.00000
REG_CITY_NOT_WORK_CITY	A flag of whether client’s permanent address matches the work address (1 = different, 0 = same, at city level)	0.00000
LIVE_CITY_NOT_WORK_CITY	A flag of whether client’s contact address matches the work address (1 = different, 0 = same, at city level)	0.00000
FLAG_DOCUMENT_3	Did client provide document 3	0.00000
AMT_ANNUITY	Loan annuity	0.00022
AMT_CREDIT	Credit amount of the loan	0.00000
AMT_GOODS_PRICE	For consumer loans it is the price of the goods for which the loan is given	0.00000
EXT_SOURCE_1	Normalized score from external data source	0.00000
EXT_SOURCE_2	Normalized score from external data source	0.00000
EXT_SOURCE_3	Normalized score from external data source	0.00019
APPS_ANNUITY_CREDIT_RATIO	Ratio of AMT_ANNUITY / AMT_CREDIT	0.00000
AVG_EXT_SOURCE	Average of EXT_SOURCE_1, EXT_SOURCE_2, EXT_SOURCE_3	0.00000

This study employs three credit scoring modelling techniques: XGBoost, LightGBM, and CatBoost. These models have been widely utilized in previous studies, such as [11,15], on the Kaggle home credit data, enabling a comparison of the models’ performance with those of prior research. Each technique is applied to develop a model using the complete set of predictor variables, after eliminating non-predictive variables. Additionally, an evaluation is conducted by excluding the 22 alternative predictor variables and reconstructing the models with the remaining predictor variables. This assessment aims to determine whether the exclusion of alternative predictor variables impacts the predictive performance of the credit models.

Furthermore, the Wald test was utilized to test the significance of the 22 alternative variables in predicting default. The Wald test is commonly used to assess the significance of predictor variables [58]. A p-value of 5% or less indicates that the predictor variable is statistically significant [58]. All predictor variables listed in Table 1 underwent the Wald test, and all p-values were found to be less than 5%, indicating their significance in the study.

### Data processing

Initially, there were 217 predictor variables. However, after applying mean, summation, maximum, and minimum aggregations on numeric features from diverse datasets, including bureau data, insights into credit history were extracted for both active and closed accounts. Numeric aggregations on application data differentiated between approved and refused applications. Additional aggregations captured transaction patterns, and instalment payment data was condensed to reflect timely payment behaviours. This increased the number of predictor variables from 217 to 767. These transformations adhere to standard feature engineering techniques, as highlighted in [36].

In many situations, predictor variables often exhibit varying minimum and maximum scales [59]. To address this issue and ensure that these variables are on a consistent scale, the technique of min-max normalization is frequently applied [59]. Min-max normalization transforms the variables, rescaling their values to fall within the range of 0 to 1 [59]. By doing so, this data preprocessing method aids in standardizing the scales of predictor variables, allowing machine learning models to better capture and understand complex relationships among them [60]. This standardization ensures that no single variable dominates the model due to differences in their scales, thus promoting a more equitable influence of all variables in the modelling process. All the 767 predictor variables have therefore been normalized to fall within the range 0 to 1.

When dealing with a large number of predictor variables in credit scoring, managing dimensionality becomes crucial [10]. While the aggregation of predictor variables is important in augmenting the variable count by creating new ones that extract customer behaviour [37], simultaneous emphasis on identifying and eliminating redundant variables is necessary. The abundance of predictor variables can lead to reduced model accuracy due to overfitting and increased model complexity [10]. Therefore, an approach to trimming predictor variables to mitigate the risk of potential information loss is necessary [61]. This delicate balance ensures nuanced and effective handling of predictor variables in credit scoring.

Some of the numerical variables exhibit missing values, ranging from 0.00046% to 80.05% of the total number of records. The social-related variables have the lowest percentage of missing input values, with the highest percentage of missing values being 0.33%. Credit bureau variables account for the highest percentage of missing values. To address these missing values, the numerical variables have been imputed with the mean of the non-missing values for each respective variable. According to the study by [62], this imputation technique is one of the most common and effective methods for handling missing values.

The variables in the research are numerical, and a challenge associated with this type of data is the presence of outliers. Outliers are observations in the data that deviate excessively from the rest of the data [63]. To address outliers, [63] recommended setting the lower and upper values of all observations in a variable to the values at the 2.5th and 97.5th percentiles, respectively. This research adopted the methodology proposed by [63] to handle outliers in the data.

Prior to applying the model-X knockoffs framework, [54] suggested incorporating a data preprocessing step to identify representative predictor variables, especially those exhibiting high correlation. This recommendation aligns with similar suggestions by other researchers, such as [64,65], emphasizing its role in reducing the dimensionality of the data. Following the guidance of [54], we applied a correlation coefficient threshold of 0.7 to assist in identifying correlated groups of predictor variables. This resulted in the formation of 551 groups of correlated predictor variables, each exhibiting a correlation coefficient of at least 0.7. Following this procedure, a representative predictor variable for each group should be selected to reduce the dimensionality of the data [54].

To identify a representative within the 551 groups of correlated predictor variables, this study adopted the approach outlined in the study by [11], employing a LightGBM model to rank and identify predictive variables within each group based on the gain metric evaluation. Within each of the 551 groups, the predictor variable with the highest gain metric is selected as the representative for that particular group.

The gain metric is a valuable approach for identifying predictive variables [66]. Gain-based feature importance assesses the significance of features in reducing impurity during tree construction, essentially evaluating the impurity difference between parent and child nodes [66]. When cumulative gains are high, it signifies a greater degree of importance [66]. Leveraging the gain metric offers advantages such as assisting in feature selection and emphasizing pivotal variables for enhancing model accuracy [46]. Nevertheless, it is important to note that the efficacy of this approach may vary based on the algorithm and hyperparameters employed in the model [67]. As a result of this step, 230 redundant predictor variables were removed, leaving a total of 321 predictor variables.

### Predictor variables selection

Before fitting the models, this study utilizes the model-X knockoffs framework, specifically employing the deep knockoffs method proposed by [54], to identify predictor variables. This application of deep knockoffs leads to a reduction in predictor variables from 321 to 215. Following this, each model is constructed using these 215 predictor variables. To assess the impact of additional predictor variables categorized as "alternative", this study conducts a comparison by excluding the alternative predictor variables listed in Table 1. As a result, the second iteration of our models is based on 193 predictor variables, excluding the alternative variables.

### Hyperparameter tuning

Achieving superior model outcomes heavily relies on precisely fine-tuning model parameters, making the hyperparameter tuning process a vital aspect of optimization [68]. In this study, the grid search technique is adopted to identify optimal parameters for all three models. Grid search is widely acknowledged for its effectiveness in determining the best hyperparameters in machine learning models [69].

### Model validation

To validate the models, the study employed a k-fold cross-validation process, a commonly used method to estimate the performance of machine learning models [70]. In k-fold cross-validation, the dataset is divided into k subsets [71], with each subset used once as the validation set while the remaining k-1 subsets are used for training. This process is repeated k times, and performance metrics are averaged across folds [71]. While providing a robust estimate of model performance, k-fold cross-validation can be computationally expensive [71].

Consistent with previous studies [11,15], a 5-fold cross-validation approach was utilized, and the reported results are based on the average performance estimates obtained from this process.

### Model performance metrics

The area under the curve metric has gained popularity in credit scoring research due to its ability to provide valuable insights into a scorecard’s discriminative power [72–74]. An AUC value greater than 0.5 indicates that the model effectively separates good and bad customers [1]. Therefore, a higher AUC score is desired as it signifies better performance in customer classification [19].

However, despite its wide use, the AUC metric does have certain limitations [75–77]. One issue arises when a credit model is poorly fitted, leading to potentially inflated or underestimated predictions of customer discrimination [77]. Additionally, interpreting the various performance thresholds provided by the AUC can be challenging for practitioners [75]. Nevertheless, despite these drawbacks, the AUC remains a prevalent and useful tool in both research and practical applications [1].

To ascertain whether differences in model performance are statistically significant, [78] introduced tests that compare the AUC of credit scorecard models [79]. This approach offers a robust method to determine if variations among models are meaningful, providing valuable insights for model selection and refinement [79].

Misclassification statistics provide a practical and interpretable tool for evaluating credit scorecard performance. This approach utilizes a confusion matrix, as depicted in Table 2, to categorize customers based on their default probability and compare their actual classifications with the scorecard’s predictions. The confusion matrix yields in four distinct cells: true negative, false positive, false negative, and true positive. In this study, analysing these cells allows for the evaluation of the accuracy of the credit scorecard predictions for both good and bad customers, as demonstrated by [19].

**Table 2:** Confusion matrix.

		**Predicted**	
		**Good**	**Bad**
**Actual**	Good	True negative	False positive
	Bad	False negative	True positive

In credit scorecard evaluation, specificity represents the accuracy of the model in predicting non-defaulting customers, while sensitivity measures its effectiveness in predicting defaulting customers. By adjusting the probability cut-off based on the scorecard’s probability of default, the aim is to minimize false positives and false negatives, striking a balance between precision and recall [19].

### Variable importance

Permutation feature importance, a technique utilized to gauge the significance of predictor variables [80], involves comparing shuffled versions of variables with their original counterparts to assess their impact on model performance. This method determines the importance of predictor variables by evaluating the model’s performance with original variable values and comparing it to performance when values are randomly rearranged. A decrease in model performance post-permutation suggests the predictor variable’s pivotal role in model accuracy, while minimal impact indicates less influence [80]. However, this technique may pose computational challenges, particularly with large datasets [81].

## Results and discussion

This section presents the outcomes of the credit scoring models and delves into their performance. This includes an in-depth examination of credit scorecards associated with each model, illustrating how the predictor variables influence the performance of the models. Through a detailed exploration of these outcomes, this section offers valuable insights into the effectiveness of the developed models.

### Performance of the models

This study began with a feature engineering process that increased the number of predictor variables from 217 to 767. To address potential correlation issues, a method from [54] was utilized to identify redundant variables. Using the gain metric, the most predictive variable within each correlated group was selected, reducing the total to 321 variables. Finally, the model-X knockoffs framework was employed to select 215 variables, including 22 alternative predictor variables related to financial, social, and geographic factors. Models were constructed using XGBoost, LightGBM, and CatBoost with and without the alternative features, allowing us to assess their impact on performance.

Table 3 presents the model performance results. Models constructed without alternative predictor variables showed reduced performance across all algorithms, as measured by AUC. The DeLong test [78] confirmed the statistical significance of these AUC differences (p-values < 0.05 for all comparisons). The LightGBM model using the full set of predictor variables achieved the highest AUC (0.79360), consistent with prior studies [11,13,14] and surpassing performance reported in previous research on this dataset. Models developed in this study also outperformed logistic regression benchmarks with AUC scores of 0.68031 [16] and 0.7574 [10].

**Table 3:** AUC of the models.

Model		p-value
XGBoost	0.78916	< 2.2e-16
XGBoost (alternative data excluded)	0.74499	
LightGBM	**0.79360**	< 2.2e-16
LightGBM (alternative data excluded)	0.75073	
CatBoost	0.78897	< 2.2e-16
CatBoost (alternative data excluded)	0.74444	

Table 4 presents the results of models trained exclusively on traditional data and models trained exclusively on alternative data. Models trained on alternative data consistently achieved higher AUC scores across all tested algorithms (XGBoost, LightGBM, and CatBoost). The DeLong test confirmed the statistical significance of these AUC improvements (p-values < 0.05). These findings provide strong evidence for the predictive power of alternative data in credit scoring, highlighting its potential to enhance model accuracy and decision-making.

**Table 4:** AUC (Traditional data vs Alternative data).

Model		p-value
XGBoost (alternative data only)	0.76378	4.925e-06
XGBoost (traditional data only)	0.74499	
LightGBM (alternative data only)	0.76177	0.008309
LightGBM (traditional data only)	0.75073	
CatBoost (alternative data only)	0.75932	0.0003902
CatBoost (traditional data only)	0.74444	

The confusion matrix in Table 5 shows that the LightGBM model (using all predictor variables) achieves the highest true negative rate (specificity) at 74.171%, while the CatBoost model has the highest true positive rate (sensitivity) at 83.459%.

**Table 5:** Confusion matrix of the three models.

**XGBoost**		**Predicted**	
		**Good**	**Bad**
**Actual**	Good	31038 (73.167%)	11383
	Bad	732	2974 (80.248%)
XGBoost (alternative data excluded)		**Predicted**	
		Good	Bad
**Actual**	Good	29841 (70.345%)	12580
	Bad	787	2919 (78.764%)
LightGBM		**Predicted**	
		Good	Bad
**Actual**	Good	31464 (74.171%)	10957
	Bad	638	3068 (82.785%)
LightGBM (alternative data excluded)		**Predicted**	
		Good	Bad
**Actual**	Good	30311 (71.453%)	12110
	Bad	711	2995 (80.815%)
CatBoost		**Predicted**	
		Good	Bad
**Actual**	Good	31368 (73.945%)	11053
	Bad	613	3093 (83.459%)
CatBoost (alternative data excluded)		**Predicted**	
		Good	Bad
**Actual**	Good	30457 (71.797%)	11964
	Bad	651	3055 (82.434%)

Table 6 shows that the LightGBM model using the full set of predictor variables achieved the lowest overall misclassification rate (25.137%).

**Table 6:** Overall misclassification rate.

Model	Overall Misclassification Rate (%)
XGBoost	26,264
XGBoost (alternative data excluded)	28,979
LightGBM	25,137
LightGBM (alternative data excluded)	27,795
CatBoost	25,291
CatBoost (alternative data excluded)	27,348

This study demonstrates the critical importance of alternative data, including financial, social, and geographic factors, for accurate credit scoring. Excluding these variables led to a significant decline in model performance.

### Performance of alternative variables

Feature importance analysis highlights the significant impact of alternative data, with variables like APPS_ANNUITY_CREDIT_RATIO, AMT_ANNUITY, and the mean of EXT_SOURCE ranking among the top predictors. This emphasizes the value of non-credit bureau attributes, such as loan structure and application details, for improving model performance. The EXT_SOURCE variables specifically demonstrate how diverse data can capture nuanced borrower behaviour.

This underscores the broader benefits of alternative data in predictive modelling. Expanding data sources improves understanding of borrowers and loans, leading to better decision-making. The inclusion of alternative variables as top predictors reinforces the need to move beyond traditional credit bureau data alone. Integrating diverse data allows for more comprehensive models and ultimately enhances risk management strategies.

## Discussion

This study demonstrates the effectiveness of the model-X knockoffs framework for variable selection in credit scoring. Incorporating alternative data sources, particularly social, geographic, financial, and behavioural variables, significantly improved model accuracy, building on prior work [6,17].

Leveraging alternative data sources in credit scoring models has several benefits. Firstly, as our study demonstrates, these models can be more predictive than those built solely on traditional data. This offers banks the opportunity to assess the creditworthiness of individuals with limited or no credit history, promoting financial inclusion. Additionally, our findings show that excluding alternative data leads to a decline in model performance across all three credit scoring models employed. This emphasizes the need for a holistic approach in credit scoring that integrates diverse sources of information.

However, the use of alternative data raises ethical concerns about privacy, potential discrimination [24,82], and the use of lifestyle factors in financial decisions. While regulations like the General Data Protection Regulation (GDPR) offer guidance, responsible implementation of these models is crucial to avoid unfair outcomes.

To transition these findings into practice, the development of scalable alternative data models must carefully balance computational costs with the need to comply with privacy and anti-discrimination regulations. Our results highlight the LightGBM model’s potential, achieving the lowest misclassification rate and demonstrating economic value for lenders. To further enhance trust and responsible use, techniques like Shapley values [82] could offer deeper insights into the impact of alternative data features without sacrificing model performance.

## Conclusion

This study expands the concept of "social scoring" [18] by examining the impact of social and geographic variables on credit risk prediction. Excluding these alternative predictors reduced model performance across all methods tested, highlighting their importance. These findings align with prior studies [20,22,31] and demonstrate the potential of alternative data for improving credit scoring models.

Using the model-X knockoffs framework for variable selection, the LightGBM model achieved the highest reported AUC (0.79360) on the Kaggle Home Credit dataset. This emphasizes the framework’s effectiveness for handling diverse data. Moreover, models trained on alternative data consistently achieved higher AUC scores across all tested algorithms (XGBoost, LightGBM, and CatBoost), with improvements confirmed as statistically significant by the DeLong test (p-values < 0.05). These findings provide strong evidence for the predictive power of alternative data in credit scoring, highlighting its potential to enhance model accuracy and decision-making.

Future research should investigate advanced feature engineering techniques specifically tailored to alternative data sources like telecom records and social media. There is also a need for streamlined feature reduction techniques that enhance model interpretability. Additionally, studies combining both credit bureau and alternative data could further illuminate the specific value of alternative variables. Evaluating the impact of alternative data on misclassification could highlight practical benefits for lenders.

Furthermore, to address concerns and advance responsible use of alternative data, future research should prioritize both privacy protection and algorithmic fairness. Techniques like differential privacy offer ways to derive insights from alternative data while safeguarding individual privacy [83]. Alongside this, research into bias mitigation algorithms and fairness assessment methods, such as counterfactual fairness testing, is crucial to ensure that models do not perpetuate or amplify societal biases [84]. This multi-pronged research direction would enable the development of credit scoring models that are both predictive and uphold principles of privacy, fairness, and non-discrimination.
